# Effect of Metformin on Testosterone Levels in Male Patients With Type 2 Diabetes Mellitus Treated With Insulin

**DOI:** 10.3389/fendo.2021.813067

**Published:** 2021-12-24

**Authors:** Tingting Cai, Yun Hu, Bo Ding, Rengna Yan, Bingli Liu, Ling Cai, Ting Jing, Lanlan Jiang, Xiaojing Xie, Yuming Wang, Huiying Wang, Yunting Zhou, Ke He, Lan Xu, Liang Chen, Cheng Cheng, Jianhua Ma

**Affiliations:** ^1^ Department of Endocrinology, Nanjing First Hospital, Nanjing Medical University, Nanjing, China; ^2^ Department of Endocrinology, Wuxi Hospital of Traditional Chinese Medicine, Wuxi, China; ^3^ Department of Endocrinology, Wuxi People’s Hospital Affiliated to Nanjing Medical University, Wuxi, China; ^4^ Department of Endocrinology, Huai’an Second People’s Hospital and The Affiliated Huai’an Hospital of Xuzhou Medical University, Huai’an, China; ^5^ Department of Endocrinology, The Affiliated Suqian First People’s Hospital of Nanjing Medical University, Suqian, China

**Keywords:** type 2 diabetic mellitus, metformin, testosterone levels, Glycated Albumin, blood glucose

## Abstract

**Aim:**

To explore the chronic effects of metformin on testosterone levels in men with type 2 diabetes mellitus (T2DM).

**Methods:**

This is a secondary analysis of a real-world study evaluating the efficacy and safety of premixed insulin treatment in patients with T2DM *via* 3-month intermittent flash glucose monitoring. Male patients aged 18-60 who were using metformin during the 3-month study period were included as the metformin group. The control group included males without metformin therapy by propensity score matching analysis with age as a covariate. Testosterone levels were measured at baseline and after 3-month treatment.

**Results:**

After 3-month treatment, the control group had higher levels of total testosterone, free and bioavailable testosterone than those at baseline (P<0.05). Compared with the control group, the change of total (-0.82 ± 0.59 vs. 0.99 ± 0.59 nmol/L) and bioavailable (-0.13 ± 0.16 vs. 0.36 ± 0.16 nmol/L) testosterone levels in the metformin group significantly decreased (P=0.036 and 0.029, respectively). In Glycated Albumin (GA) improved subgroup, the TT, FT, and Bio-T levels in the control subgroup were higher than their baseline levels (P < 0.05). Compared with the metformin subgroup, TT level in the control subgroup also increased significantly (P=0.044). In GA unimproved subgroup, the change of TT level in the metformin subgroup was significantly lower than that in the control subgroup (P=0.040).

**Conclusion:**

In men with T2DM, 3-month metformin therapy can reduce testosterone levels, and counteract the testosterone elevation that accompanied with the improvement of blood glucose.

**Clinical Trial Registration:**

https://www.clinicaltrials.gov/ct2/show/NCT04847219?term=04847219&draw=2&rank=1.

## Introduction

Type 2 diabetes mellitus (T2DM) is a common metabolic disease characterized by hyperglycemia and insulin resistance, which can affect the normal function of the whole body, lead to cardiomyopathy, atherosclerosis, nephropathy and peripheral neuropathy, and increase the risk of neurodegenerative and other endocrine diseases (1, 2). It not only affects the quality of life and survival of patients, but also brings substantial physiological and psychological burden to patients (3).

Testosterone, the main androgen in the male reproductive process, is responsible for the development of secondary sexual characteristics, sexual desire, and erectile function (4). Normal testosterone level in men is essential to maintain bone mineral density, muscle growth, brain nervous system and cognitive health (5, 6). It is reported that low testosterone is widespread in men with metabolic syndrome such as T2DM and obesity. About one-third of T2DM men have hypogonadism (7, 8). The reason may be due to long-term hyperglycemia, resulting in metabolic imbalance, inflammation, and oxidative stress (9). There is increasing evidence that men with low testosterone levels have lower survival rates and higher all-cause, cardiovascular, cancer, and respiratory mortality rates than men with high or normal testosterone levels (10, 11). Therefore, we should pay attention to the change of testosterone level in patients with T2DM. Metformin is a first-line drug widely used in the treatment of T2DM. It is a stable, low molecular weight hydrophilic compound, which can reach a variety of tissues, including muscle, liver, pancreas, adipose tissue, hypothalamus, pituitary, and gonad (12). In addition to the treatment of T2DM, metformin also plays some other beneficial effects. Recent data have demonstrated the advantageous effects of metformin in cancer, cardiovascular disease, and polycystic ovary syndrome (13, 14).

Previous study found that patients with T2DM had significantly lower testosterone levels after one month of metformin treatment (15). In the previous study, patients experienced rapid normalization of blood glucose using insulin therapy within 5 days and followed with a short term of metformin therapy for one month. Therefore, to exclude the influence of the rapid change of blood glucose before metformin therapy and further explore the chronic effect of metformin treatment on testosterone levels in male patients with T2DM, this study observed testosterone levels in male patients with T2DM who had stable insulin therapy for at least 2 months, and prolonged metformin treatment for another 3 months.

## Methods

### Study Design and Participants

Our study is a secondary analysis of a premix insulin study (ClinicalTrials.gov NCT 04847219, Supplementary file), which was conducted in the outpatient department of endocrinology of five hospitals in Jiangsu Province from October 2019 to April 2021. The study was approved by ethics committee of Nanjing First Hospital. All operations were in accordance with the ethical standards of the hospital and the 1964 Helsinki Declaration revised in 2013. All participants obtained informed consent.

The inclusion criteria were as follows: 1) patients aged >18 years and diagnosed with T2DM according to World Health Organization 1999 diagnostic criteria of T2DM; 2) patients using subcutaneous injection with premix insulin Bid/Tid, single drug and/or combination of oral hypoglycemic drugs, the treatment regimen was stable for more than 2 months; 3) subjects were willing to undergo Flash Glucose Mornitoring (FGM) examination;

The exclusion criteria were as follows: 1) patients treated with GLP-1 agonist or systemic hormone therapy in recent 3 months; 2) patients with insulin allergy or FGM intolerance; 3) impaired liver and renal function, ALT 2.5 times higher than the upper limit of normal value; serum creatinine was 1.3 times higher than the upper limit of normal; 4) patients with acute metabolic diabetic complications, infection, stress or any other apparent condition as determined by the investigator (e.g., severe heart and lung disease, endocrine disease, neurological disease, tumor disease, other pancreatic disease, history of mental illness).

All eligible subjects received intermittent FGM once a month for 3 months. Doctors adjusted the hypoglycemia treatment, and diabetes specialist nurses provided educations about insulin injection techniques, self-management of diet and exercise according to the FGM data every month.

Based on this study, we selected male patients aged 18-60 who were treated with metformin for three months during the premixed insulin study as the metformin group (n=40). The mean dose of metformin (Merck Serono) was 1500 mg daily. The control group was selected from the remaining male patients who were not treated with metformin through propensity score matching adjusted for age (n=40).

Data such as age, weight, insulin dose and oral hypoglycemic drugs were collected at baseline and end point of study. Biochemical parameters such as fast C-peptide and fast insulin were measured by routine laboratory methods at baseline and end point of the study. Glycated hemoglobin (HbA1c) was determined by high performance liquid chromatography (Bio-Rad, Diastat HbA1c analyzer). Glycated Albumin (GA) was determined by peroxidase method (Jiuqiang biological kit). Total testosterone (TT) and sex hormone binding globulin (SHBG) were measured by chemiluminescence (Unicel of Beckman Coulter, USA™ DXI 800 automatic analyzer). Free testosterone (FT) and bioavailable testosterone (Bio-T) were calculated (16). The change of testosterone levels was the main observation index of this study.

### Statistical Analysis

Spss23.0 software (SPSS, IL, USA) was used for statistical analysis. The data of normal distribution are expressed as average ± standard error and nonnormal distribution data are represented by median in the quartile range. Enumeration data was analyzed by chi-square test (the proportion of hypoglycemic drugs and percentage of subjects with TT<12nmol/L). The data before and after treatment were analyzed by Student paired t-test or Wilcoxon test. Covariance analysis was used to analyze the differences in testosterone levels between groups, and insulin dose was used as a covariate. The significance level was 5%.

## Results

### Baseline Characteristics

A total of 40 male patients were enrolled in the metformin group, and another 40 males who did not use metformin were matched as controls. Finally, 80 people were included in the analysis of this study. According to International Society for Sexual Medicine, International Society for Sexual Medicine (ISSM) guidelines, men with TT < 12nmo/L have a lack of testosterone levels (17). Low serum testosterone usually indicates hypogonadism. All characteristics were not significantly different between the two groups at baseline (P all >0.05, **Table 1**).

**Table 1:** Baseline clinical and laboratory characteristics of the study participants.

Baseline	Metformin	Control	P-Value
N	40	40	
Age (yrs.)	63.78 ± 1.40	62.00 ± 1.78	0.437
Weight (kg)	72.84 ± 1.31	72.18 ± 1.61	0.752
BMI (kg/m^2^)	25.86 ± 0.38	25.02 ± 0.51	0.191
HbA1c (%)	7.72 ± 0.22	7.27 ± 0.16	0.102
GA (%)	18.83 (16.29, 21.39)	18.9 (16.28, 23.89)	0.889
Fast C-peptide (ng/ml)	0.95 (0.7, 1.86)	1.28 (0.71, 2.00)	0.973
Fast insulin (pmol/l)	13.7 (8.6, 26.05)	11.9 (7.85, 24.1)	0.548
Insulin dose (IU/day)	39.23 ± 2.11	36.40 ± 2.19	0.356
Acarbose (%)	11 (27.5%)	10 (25%)	1
Insulin secretagogues (%)	3 (7.5%)	2 (5%)	1
DPP-4 inhibitors (%)	4 (10%)	2 (5%)	0.675
TZDs (%)	1 (2.5%)	1 (2.5%)	1
TT<12 nmol/l (%)	9 (22.5%)	10 (25%)	0.785
SHBG (nmol/l)	38.07 ± 2.04	40.97 ± 2.88	0.414
TT (nmol/l)	15.91 ± 0.79	15.71 ± 0.88	0.874
FT (nmol/l)	0.24 ± 0.01	0.24 ± 0.01	0.736
Bio-T (nmol/l)	4.69 ± 0.19	4.64 ± 0.20	0.858

Data were presented as means ± SE or median (25th, 75th percentile). BMI, body mass index; HbA1c, glycated hemoglobin; GA, Glycated Albumin; SHBG, sex hormone binding globulin; TT, total testosterone; FT, Free testosterone; Bio-T, bioavailable testosterone;

### The changes of Testosterone Levels in the Two Groups

**Figure 1:**
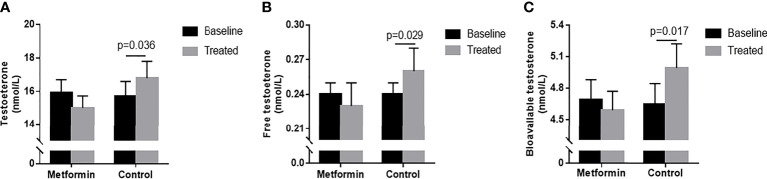
**(A–C)** TT, FT and Bio-T levels at baseline and endpoint in metformin and control group. Data are mean ± SE.

TT, FT, and Bio-T levels significantly increased after 3-month in the control group. (15.71 ± 0.88 vs. 16.79 ± 1.01 nmol/L, 0.25 ± 0.02 vs. 0.27 ± 0.02 nmol/L, 4.89 ± 0.23 vs. 5.21 ± 0.24 nmol/L, P all<0.05, **Figures 1A–C**), but the change was lost in metformin group (P all >0.05).

**Figure 2:**
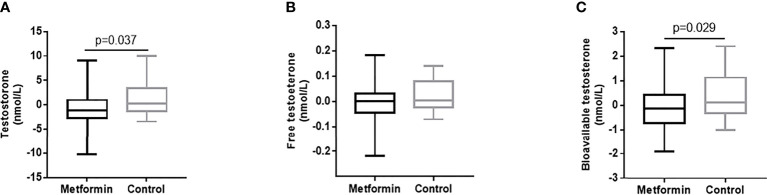
**(A–C)** The changes of TT, FT and Bio-T levels between metformin and control group before and after treatment. Data are mean ± SE.

The change of testosterone levels = TT level after treatment -TT level before treatment. Patients in metformin group caused superior reductions in TT and Bio-T versus the control group (estimated treatment difference: -1.81 95%CI [-3.507, -0.112], -0.49 95%CI [-0.929, -0.051], P=0.037 and 0.029, respectively, **Figures 2A, C**). There was no significant difference found in the change of FT between two groups. (**Figure 2B**)

### The Changes of Testosterone Levels Stratified by Glycated Albumin

**Figure 3:**
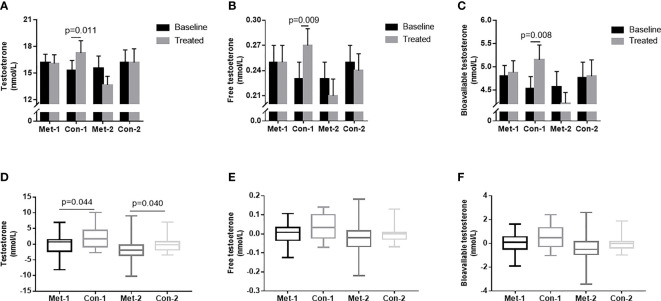
**(A–C)** TT, FT and Bio-T levels at baseline and endpoint in metformin and control groups under different stratification. **(D–F)** the changes of TT, FT and Bio-T levels between metformin and control group before and after treatment under different stratification. 1=△GA ≤ 0; 2 =△GA>0; Data are mean ± SE.

To explore the influence of blood glucose changes on the testosterone level of patients, we subdivided the patients into four groups according to changes in GA: GA improved (endpoint GA– baseline GA ≤ 0) metformin group (metformin-1 group, n=22); GA improved control group (control-1 group, n=22); GA unimproved (endpoint GA– baseline GA >0) group (metformin -2 group, n=18); GA unimproved control group (control-2 group, n=18). With 3-month treatment, the control-1 group had higher levels of TT, FT, and Bio-T than those at baseline (P all <0.05, **Figures 3A–C**). There was no statistically significant difference in TT, FT, and Bio-T levels among the other three groups before and after treatment (P all >0.05, **Figures 3A–C**). The change of TT level in the control group 1 was higher than that in the metformin group (P=0.044, **Figure 3D**), and the change in the metformin group 2 was significantly lower than that in the control group 2 (P=0.040, **Figure 3D**). As shown in **Figures 3E, F**, there was no difference between groups in the changes of FT and Bio-T.

### Endpoint Characteristics

At the endpoint, the insulin dose used in the metformin group was lower than that in the control group (P=0.004). There was no significant difference between the two groups in the parameters that could affect testosterone levels, such as body weight HbA1C, GA, fast C-peptide, fast insulin, TT<12nmol/L (%) and other oral drugs between the two groups (P all >0.05, **Table 2**).

**Table 2:** Endpoint characteristics of the study participants.

Endpoint	Metformin	Control	P-Value
Weight (kg)	72.69 ± 1.24	72.20 ± 1.24	0.805
HbA1c (%)	7.33 ± 0.17	7.09 ± 0.15	0.304
GA (%)	18.99 (15.93, 22.39)	19.28 (16.67, 22.49)	0.560
Fast C-peptide (ng/ml)	1.20 (0.79, 1.86)	1.32 (0.80, 1.90)	0.617
Fast insulin (pmol/l)	13.80 (8.78, 28.70)	12.45 (8.05, 24.48)	0.450
Insulin dose (IU/day)	34.50 (28.50, 44.50)	38.75 (26.00, 38.75)	0.004*
Acarbose (%)	12 (30%)	8 (20%)	0.439
Insulin secretagogues (%)	1 (2.5%)	3 (7.5%)	0.615
DPP-4 inhibitors (%)	11 (27.5%)	11 (27.5%)	1
TZDs (%)	0 (0%)	3 (7.5%)	0.241
TT<12 nmol/l (%)	8 (20%)	11 (27.5%)	0.431

Data were presented as means ± SE or median (25th, 75th percentile). HbA1c, glycated hemoglobin; GA, Glycated Albumin; *, Metformin vs. Control, p<0.05

## Discussion

This real-word study reveals that 3-month metformin therapy can reduce testosterone levels, and counteract the testosterone elevation that accompanied with the improvement of blood glucose in men with T2DM.

In men, there is strong evidence that low testosterone levels are significantly associated with T2DM (18). The mechanism remains unclear. One reason may be the pro-inflammatory cytokines induced by hyperglycemia (19). Proinflammatory cytokines can inhibit the secretion of gonadotropin-releasing hormones, thereby reducing the level of circulating luteinizing hormone *in vivo* and *in vitro (* 20), indicating that a pro-inflammatory state may contribute to the central inhibition of the male sex axis. Intensive insulin therapy can reduce the inflammatory response (21). Previous studies have also found that short-term intensive therapy for 5 days can significantly increase the level of testosterone (15). In the present study, improvement of glycemic control increased the testosterone level in a longer term of 3 months, which confirmed the effects of hyperglycemia on sexual hormones.

Animal studies have found that metformin can improve the testicular function and spermatogenesis of obese male mice induced by high-fat and high-cholesterol diet (22), and restore the gonadotropin and leptin systems in the testes of streptomycin-induced male rats (23). However, given the complex between glycemic control and hormone, these studies did not rule out the effect of blood glucose changes on it. Our previous study showed that metformin therapy can reduce testosterone levels in males with T2DM who had normalized blood control (15). This finding indicated that the use of metformin may be another reason of the high prevalence of low testosterone in males with T2DM. The present study prolonged the duration of metformin therapy, and the results were consistent with the previous study. Moreover, our stratification analysis with the change of GA indicated that the effects of metformin on testosterone levels existed regardless of the change of blood glucose. Combined with the results of the two studies, the effect of metformin on testosterone levels was independent of blood glucose.

M Faure *et al. (* 24) found that metformin exposure *in vitro* may contribute to the decrease of cell proliferation and the change in secretory ability of testicular Sertoli cells. *In vivo*, metformin exposure negatively affected the germ cell population. Metformin exposure resulted in a decrease in testicular weight and sperm cell production in chickens, suggesting that taking metformin in drinking water for 3 weeks was sufficient to delay spermatogenesis. Sertoli cells are well known to regulate the synthesis and secretion of testosterone by Leydig cells (25). This may contribute to the reduction of testosterone by metformin. However, additional studies are needed to clarify the mechanisms that metformin reduces testosterone in male patients with T2DM.

Although previous studies have found that testosterone levels are negatively correlated with insulin resistance (26, 27), testosterone replacement therapy can reduce insulin resistance (28). In this study, although the testosterone level of the metformin group decreased, the dose of insulin injection was reduced, and the blood glucose of the two groups were similar, indicating that the level of insulin resistance still improved. Therefore, the use of metformin needs to be considered in the relevant research on improving insulin resistance through testosterone replacement therapy, and the relationship between testosterone changes and insulin resistance in patients using metformin still needs to be further studied.

In fact, the importance of testosterone in men with T2DM is often ignored. In 2018, the American Diabetes Association added a recommendation in the standard of diabetes medical care to measure testosterone levels in men with symptoms of diabetes and hypogonadism (29). Many studies have shown that testosterone replacement therapy can prevent prediabetes or T2DM status in men with low testosterone (30–32). Furthermore, attention should be paid to the effect of drugs on testosterone levels in men with T2DM, which is the value of this study.

Some limitations of this study deserve comment. The study population is patients using premixed insulin. Whether the research results can be extended to the whole population needs further research. Although there is a control group, the effect of changes in the dose of insulin or other hypoglycemic drugs cannot be completely excluded in the real-world study.

In conclusion, our data indicate that the 3-month metformin treatment can reduce testosterone levels in men with T2DM. In the future, attention should be paid to the effect of drugs on male testosterone levels in the treatment of diabetes.

## Data Availability Statement

The raw data supporting the conclusions of this article will be made available by the authors, without undue reservation.

## Ethics Statement

The studies involving human participants were reviewed and approved by ethics committee of Nanjing First Hospital. The patients/participants provided their written informed consent to participate in this study.

## Author Contributions

TC and YH performed, analyzed data and wrote the manuscript. BD, RY, BL, LCa, TJ, LJ, YW, HW, YZ, KH, LX, LCh, CC organized data. XX modified the manuscript. JM conceived, and directed the study. All authors contributed to the article and approved the submitted version.

## Funding

This study was partly supported by the National Key R&D Program of China (No. 2018YFC1314103).

## Conflict of Interest

The authors declare that the research was conducted in the absence of any commercial or financial relationships that could be construed as a potential conflict of interest.

## Publisher’s Note

All claims expressed in this article are solely those of the authors and do not necessarily represent those of their affiliated organizations, or those of the publisher, the editors and the reviewers. Any product that may be evaluated in this article, or claim that may be made by its manufacturer, is not guaranteed or endorsed by the publisher.
